# Genome-wide identification of the GLK transcription factor family in melon and its expression analysis under biotic and abiotic stresses

**DOI:** 10.3389/fpls.2025.1679452

**Published:** 2025-10-29

**Authors:** Ling Zheng, Yuna Wang, Fang Lv, Jianming Han

**Affiliations:** Department of Biology, Luoyang Normal University, Luoyang, Henan, China

**Keywords:** melon, *GLK* gene family, *Fusarium* wilt stress, drought stress, expression analysis, bioinformatics

## Abstract

This study systematically identifies and characterizes the *GLK* gene family in melon (*Cucumis melo*), identifying 49 *GLK* genes within the melon genome, which are subsequently named *CmGLK1* to *CmGLK49* based on their chromosomal locations. The *CmGLK* family members exhibit significant variation in physicochemical properties, including amino acid length, molecular weight, and isoelectric point. Phylogenetic analysis classified the *CmGLK* genes into six groups (I-VI), demonstrating a high degree of conservation of *GLK* genes across melon, cucumber, and watermelon. Motif and domain analyses indicate that all *CmGLK* family members possess the characteristic *GLK* transcription factor domain. Moreover, genes within the same phylogenetic group display similar protein motifs and gene structures, suggesting that these genes may have a common function in regulating plant growth and development. Chromosomal localization reveals that *CmGLK* genes are unevenly distributed across 12 chromosomes, with nine pairs of segmental duplications and one pair of tandem repeats, indicating that gene duplication likely plays a key role in the expansion of this gene family. Additionally, *GLK* homologs were identified between melon and *Arabidopsis thaliana*, *Oryza sativa*, *Cucumis sativus*, and *Citrullus lanatus*, with the highest homology found between melon and cucumber, as well as melon and watermelon. *Cis*-regulatory element analysis of *CmGLK* promoters reveals a high abundance of elements associated with light response, hormone regulation, and stress responses, suggesting that the *CmGLK* family may be involved in the regulation of melon growth, development, and environmental stress responses. Expression profiling shows that *CmGLK* genes are expressed in a tissue-specific manner, with the highest expression levels found in the root, stem, and leaf tissues. Further Quantitative fluorescence analysis reveals that several *CmGLK* family members exhibit significant expression changes under *Fusarium* wilt and drought stress, suggesting their potential involvement in melon’s stress response mechanisms. This study provides a comprehensive analysis of the structure, evolution, and stress-responsive expression of the *CmGLK* gene family. Our findings offer new insights into the potential molecular mechanisms of melon’s stress adaptation and highlight candidate *CmGLK* genes for future functional studies aimed at improving disease resistance.

## 1 Introduction

Chloroplasts are essential organelles in plants, serving as the primary site for photosynthesis and the production of energy and metabolites. Their proper formation is crucial for plant growth and development (Razi and Muneer, 2021). As semi-autonomous organelles, chloroplasts can independently replicate DNA, transcribe, and translate a portion of the proteins they require. However, they are also tightly regulated by nuclear-encoded gene products (Mayfield et al., 1995). Among these gene products, the GOLDEN2-LIKE (GLK or G2-like) transcription factors play a key role in chloroplast formation, development, and senescence. The coordination between nuclear and chloroplast genes ensures proper chloroplast development, thereby optimizing photosynthesis in plants (Shen et al., 2022).

The *GLK* gene family was first identified in genes responsible for yellowing in maize (Hall et al., 1998) and is classified as part of the GARP superfamily (Riechmann et al., 2000). Typical *GLK* transcription factors contain two conserved domains: a Myb-DNA binding domain (DBD) and a C-terminal domain with a conserved GCT-box (Rossini et al., 2001). The Myb-DNA binding domain includes a helix-loop-helix (HLH) structure, where the first helix contains the PELHRR motif, the second helix contains the NI/VASHLQ motif, and the loop connecting the helices consists of 22 amino acids (Liu et al., 2016).

Existing studies have demonstrated that *GLK* transcription factors are primarily involved in regulating processes such as chloroplast development, senescence, and chlorophyll accumulation (Fitter et al., 2010). For instance, *GLK1* and *GLK2* are critical for chloroplast formation and development in *A. thaliana* (Kobayashi et al., 2013; Waters et al., 2009). Moreover, *GLK* family members are associated with responses to both biotic and abiotic stresses. Through interactions with other factors, *GLK* transcription factors regulate the expression of resistance-related genes, thereby enhancing photosynthesis under stress conditions (Han et al., 2016). For example, under fungal or viral infections, *A. thaliana* shows a significant increase in *GLK*gene expression, and overexpression of *GLK1* enhances resistance to *Fusarium graminearum* (Savitch et al., 2007). Transformation of maize *GLK* genes into rice reduces photoinhibition and improves CO_2_ fixation efficiency under high-light conditions (Xia et al., 2020). These findings suggest that *GLK* genes may play an important role in stress tolerance. Reports have also indicated that *GLK1* and *GLK2* in the *GLK* gene family are differentially involved in chloroplast development in C_3_ and C_4_ photosynthetic plants (Peng et al., 2017). In maize, *ZmGLK1* is predominantly expressed in mesophyll cells, while *ZmGLK2* exhibits stronger expression in bundle sheath cells, indicating spatial compartmentalization essential for chloroplast development in these cell types (Chang et al., 2012). In tomato, *SlGLK1* and *SlGLK2* enhance photosynthesis in fruits and increase the expression of chloroplast development genes, leading to higher carbohydrate and carotenoid content in mature fruits, which provides greater resistance to cold, drought, and heat stress (Nguyen et al., 2014).

Melon (*C. melo*), commonly known as “honeydew melon” or “muskmelon,” is a rich source of glucose, minerals, and vitamins, providing health benefits such as cooling, nutritional supplementation, and diuretic effects (Gao, 2015). However, the yield and quality of melons are influenced by various factors, including genetic traits, cultivation practices, and environmental stressors. *F.* wilt, caused by *F. oxysporum*, is a major fungal disease that severely impacts melon production in Henan, often resulting in plant wilting and death, which adversely affects both yield and fruit quality (Herman and Perl-Treves, 2007). Moreover, drought stress in the region exacerbates the challenges associated with melon cultivation. Some studies have been conducted on the response of various gene families in melon to *F.* wilt stress (Zheng et al., 2024; 2025). Current research on the *GLK* gene family in response to biotic stress is primarily focused on *A. thaliana* (Murmu et al., 2014; Schreiber et al., 2011), while a relevant investigation in watermelon has also reported the involvement of *GLK* genes in stress responses (Rahman et al., 2021).However, there is a lack of research on the role of the *GLK* gene family in *F.* wilt stress or drought stress in melon. Bioinformatics methods were used to analyze the chromosomal localization, phylogenetic relationships, synteny, gene structure, protein motifs, expression patterns, and *cis*-regulatory elements of the melon *GLK* gene family. Furthermore, the expression of these genes under *F.* wilt (biotic Stress) and drought (abiotic Stress) stresses was examined, providing a foundation for understanding the functional roles of the *GLK* gene family in melon and offering molecular targets for its genetic improvement.

## 2 Materials and methods

### 2.1 Identification of GLK gene family in melon

The melon DHL92 v4.0 genome was downloaded from the CuGenDB database (http://cucurbitgenomics.org/) for GLK gene identification (Garcia-Mas et al., 2012). Protein sequences of 36 *A. thaliana* GLK family members were obtained from the UniProt database (https://www.uniprot.org/) (UniProt Consortium et al., 2021). Using the BLAST tool, the melon genome protein data were aligned with the *A. thaliana* GLK protein sequences, and duplicates were removed, retaining genes with an e-value ≤ e^−5^ (Ye et al., 2006). Domain identification was performed using the NCBI Conserved Domain Database (CDD), and genes containing the GOLDEN2-LIKE domain were selected. In total, 49 *GLK* genes were identified in the melon genome. The physicochemical properties of the proteins were then analyzed using ExPASy (http://web.expasy.org/protparam/) (Wilkins et al., 1999), and subcellular localization predictions were conducted using WoLF PSORT II (https://www.genscript.com/wolf-psort.html?src=leftbar) (Horton et al., 2007). Additionally, 55 GLK family members were respectively identified in the cucumber and watermelon genomes using the same method.

### 2.2 Phylogenetic tree construction and classification

Multiple sequence alignment of GLK family proteins from melon, cucumber, watermelon, and *A. thaliana* was performed using MEGA 7 software and the MUSCLE algorithm. The phylogenetic tree was constructed using the Neighbor-Joining (NJ) method with 1,000 bootstrap replicates, employing the Poisson correction model and pairwise deletion (Kumar et al., 2016). Based on the phylogenetic tree, the *GLK* family members were classified into six groups: Group I, Group II, Group III, Group IV, Group V, and Group VI.

### 2.3 Conserved motifs, domains, and gene structure

Conserved motifs in the 49 CmGLK protein sequences were identified using the MEME tool (http://meme-suite.org/tools/meme), with a maximum of 10 motifs and default parameters (Bailey et al., 2009). The positions and numbers of GLK domains in the CmGLK family proteins were determined using the NCBI CDD online tool (https://www.ncbi.nlm.nih.gov/cdd/) (Marchler-Bauer et al., 2015). The positions and numbers of exons and introns in the *CmGLK* genes were extracted from the melon genome data. Finally, the phylogenetic tree, motifs, domains, and gene structures of the *CmGLK* family members were clustered and visualized using TBtools (Chen et al., 2020).

### 2.4 Gene localization, duplication, and Ka/Ks ratio

The chromosomal distribution of *CmGLK* genes was mapped using the TBtools software. Fragment duplication and tandem repeat gene analyses were analyzed with the MCScanX software, and the chromosomal synteny map was generated with the Circos software (Wang et al., 2012; Krzywinski et al., 2009). The *GLK* orthologous gene pairs between melon and *A. thaliana*, as well as between melon and rice, were identified. The Ka (nonsynonymous substitution rate), Ks (synonymous substitution rate), and Ka/Ks ratio for all types of duplicated genes were determined with the KaKs_Calculator (Zhang et al., 2006).

### 2.5 Promoter annotation

The 2000 bp upstream promoter sequences of the 49 *CmGLK* genes were extracted and their *cis*-regulatory elements annotated using the PlantCARE online tool (http://bioinformatics.psb.ugent.be/webtools/plantcare/html/) (Lescot et al., 2002). The *cis*-regulatory elements associated with abiotic and biotic stresses, phytohormone responsiveness, and plant growth and development, as identified in previous studies, were analyzed for positional distribution (Zhang et al., 2024).

### 2.6 Expression profiling

The expression profiles of *CmGLK* family members in 13 different tissues—Root, Middle stem, Upside stem, Shoot apex, Young leaves, 12th leaves, Tendril, Anther male flower, Anther female flower, Petal female flower, Stigma female flower, Ovary DAF4, and Fruit flesh DAF50—were obtained by analyzing the FPKM (Fragments Per Kilobase of exon model per Million mapped fragments) values from the melon transcriptome database (http://cucurbitgenomics.org/rnaseq/home). Genes with an FPKM value greater than or equal to 0.8 were defined as significantly expressed (Zhang et al., 2024; Yu et al., 2023).

### 2.7 RNA extraction, cDNA synthesis, and qRT-PCR analysis of the melon GLK gene family

Seeds of the melon cultivar “Super Sweet White Sugar Melon” were soaked, germinated, and planted. The plants were grown in a greenhouse until the three-leaf and one-heart stage, with a light cycle of 16/8 hours (day/night) and temperatures of 28/18 °C (day/night). *F.* wilt was isolated from infected melon plants. A spore suspension (1 × 10^^8^ spores/mL) was applied to the plants through root irrigation, with 10 mL of suspension per plant. For the control (CK) group, the same root irrigation method was used, and each plant was irrigated with 10 mL of sterile water (distilled water) instead of the *F.* wilt spore suspension. Real-time quantitative PCR (qRT-PCR) was used to investigate the expression levels of 15 *CmGLK* genes at four time points (0, 12, 24, and 48 hours) following drought stress treatment (leaves; three biological replicates) and at four time points (0, 24, 48, and 96 hours) following *F.* wilt infection (stems; three biological replicates).

RNA quality and concentration were assessed using the Nanodrop 2000 instrument. First-strand cDNA was synthesized using the SweScript All-in-One First-Strand cDNA Synthesis Kit (TRANS, G3337). Quantitative PCR (qPCR) analysis was performed using the 2×SYBR Green qPCR Master Mix (without ROX) (TRANS, G3320) on the Bio-Rad CFX96 Real-Time PCR Detection System. XM_008449635.3 was used as the internal reference gene for data normalization. Three biological and three technical replicates were performed for each sample. The relative expression levels of the *GLK* family genes were calculated using the 2^−ΔΔCT^ method. The statistical significance of differences among treatment groups was determined by one-way ANOVA followed by the least significant difference (LSD) *post-hoc* test using SPSS 19.0, as shown in **Figures 2**, **3**.

**Figure 1:**
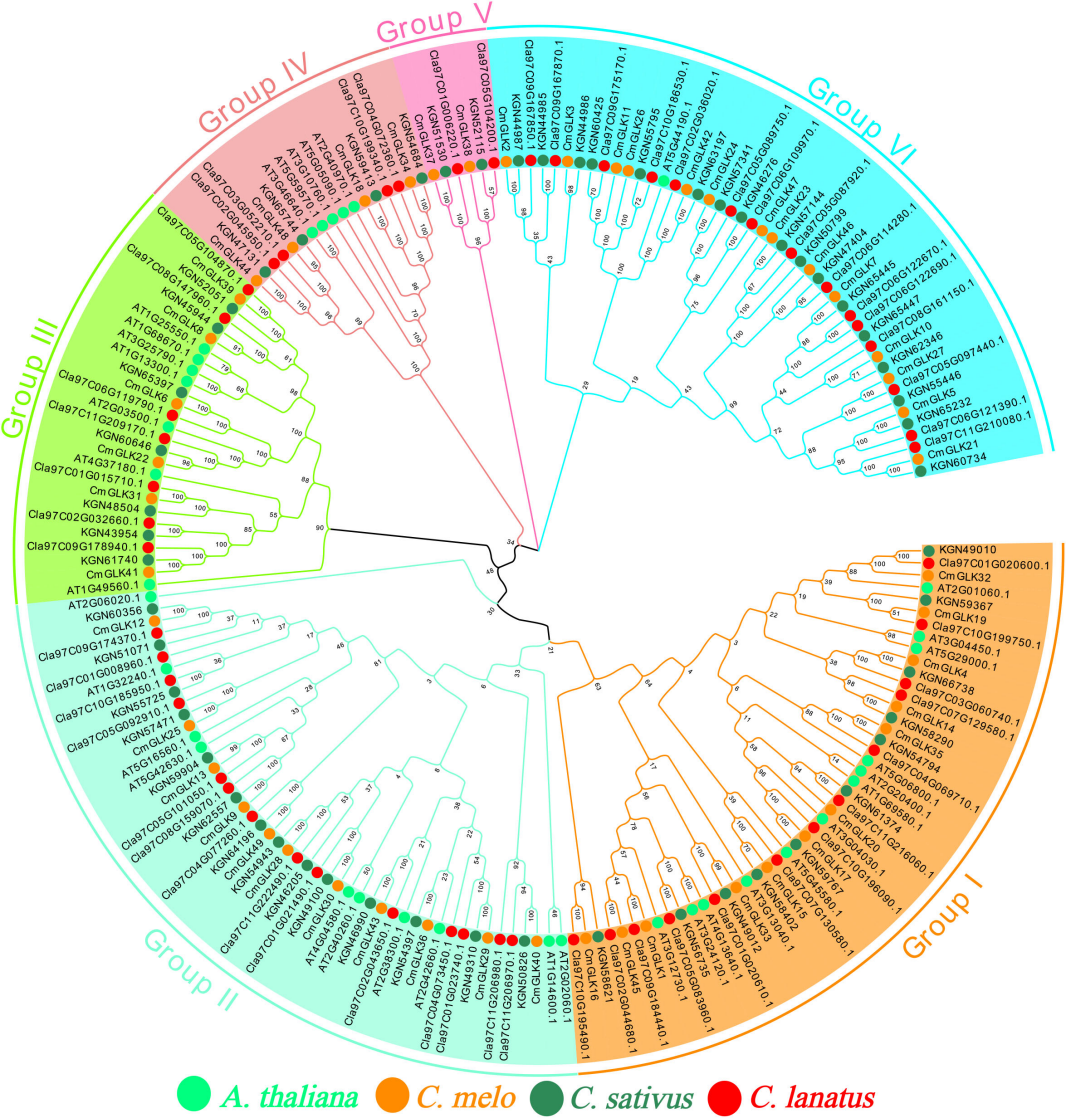
Chromosomal localization of the *CmGLK* gene family. The six different colors represent the six Groups, and the solid circles in four colors represent the *GLK* genes from four species.

**Figure 2:**
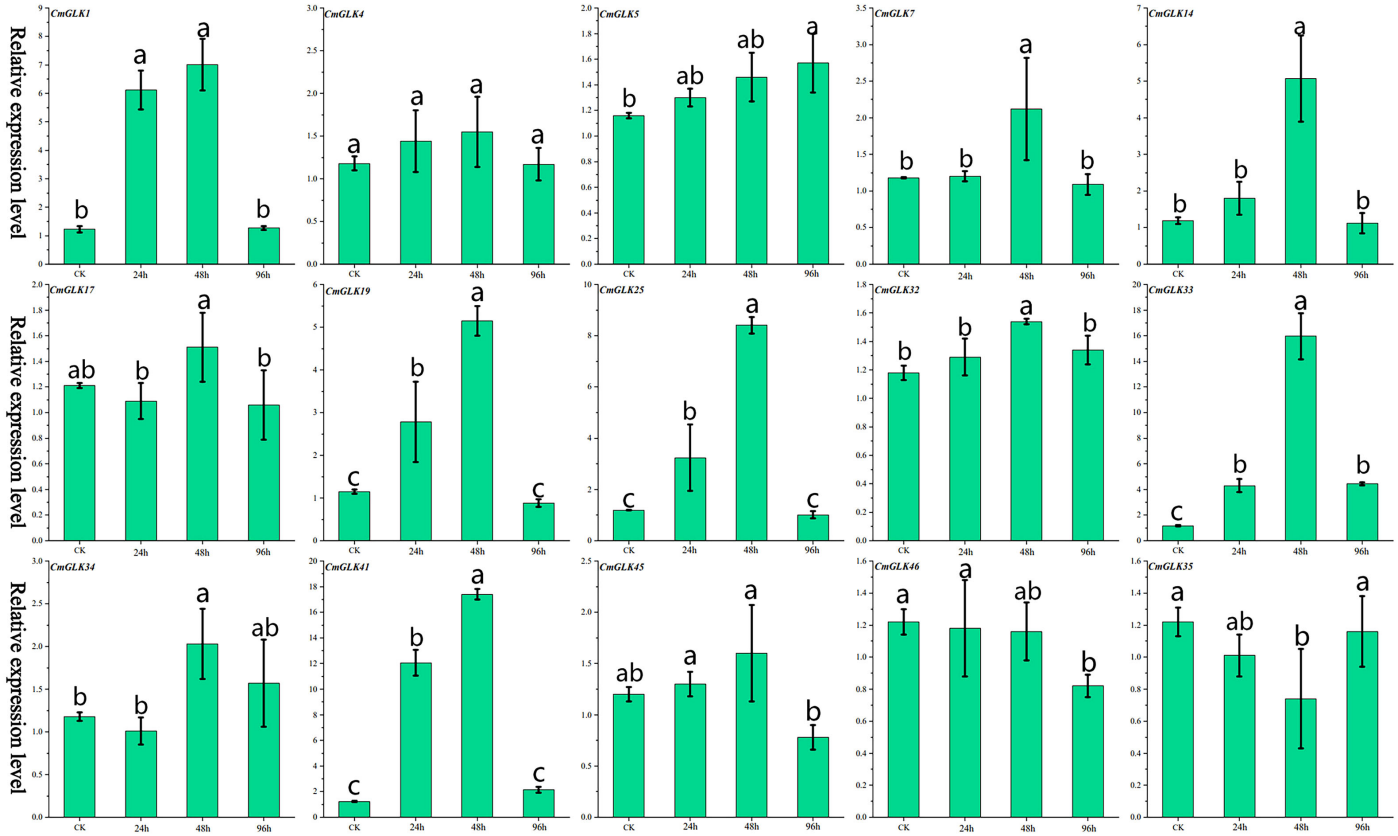
Relative expression levels of 15 *CmGLK* genes at four time points under *F.* wilt stress. Values are presented as mean ± SD (n = 3). Bars labeled with different lowercase letters indicate statistically significant differences among treatment groups (p < 0.05) as determined by one-way ANOVA followed by the least significant difference (LSD) *post-hoc* test.

**Figure 3:**
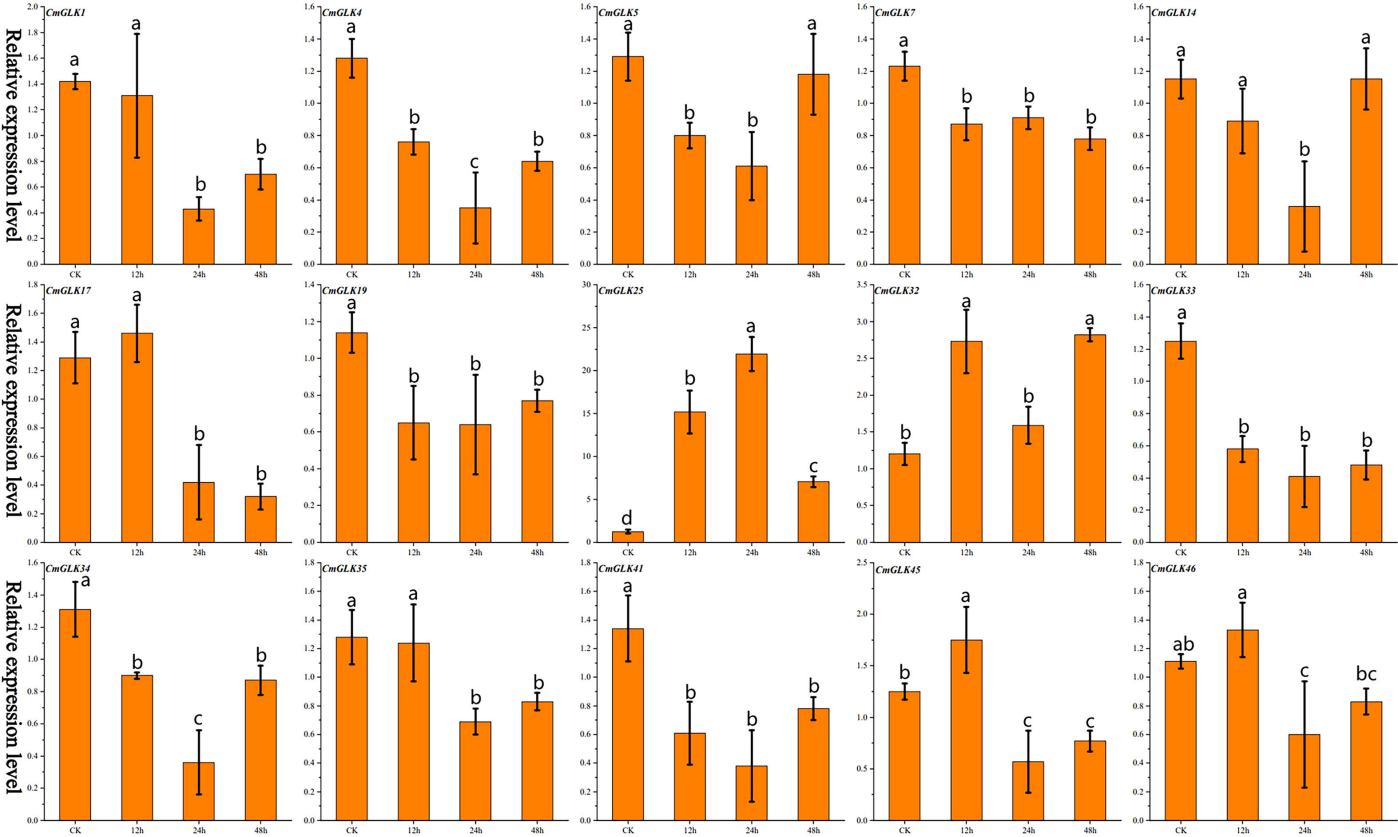
Relative expression levels of 15 *CmGLK* genes at four time points under drought stress, represented as a bar chart. Values are shown as mean ± SD (n=3).Bars labeled with different lowercase letters indicate statistically significant differences among treatment groups (p < 0.05) as determined by one-way ANOVA followed by the least significant difference (LSD) *post-hoc* test.

## 3 Results and analysis

### 3.1 Identification and analysis of the melon GLK gene family

A total of 49 *GLK* genes were identified in the melon genome, which were renamed according to their chromosomal positions as *CmGLK1* to *CmGLK49*. The physicochemical properties of the family members revealed considerable differences among them (**Supplementary Table S1**). The number of amino acids in the *CmGLK* family members ranged from 92 aa (*CmGLK20*) to 699 aa (*CmGLK21*), with protein molecular weights ranging from 10,573.15 Da (*CmGLK20*) to 76,840.49 Da (*CmGLK21*). The theoretical isoelectric points of the *CmGLK* family members ranged from 5.13 to 10.07, with 27 members having an isoelectric point below 7 and 22 members having an isoelectric point above 7. All *CmGLK* transcription factors were hydrophobic proteins. Subcellular localization analysis indicated that 47 *CmGLK* members were localized in the nucleus, while 2 were located in the mitochondria.

### 3.2 Phylogenetic analysis of CmGLK

A Neighbor-Joining phylogenetic tree of *GLK* family members from melon, cucumber, watermelon, and *A. thaliana* was constructed using the MEGA 7 tool (**Figure 1**). The tree was divided intosix groups: Group I, Group II, Group III, Group IV, Group V, and Group VI. Group I to Group VI contained 12, 11, 6, 4, 2, and 14 *CmGLK* genes, respectively. The clustering results showed that GLK family members from melon, cucumber, and watermelon were highly conserved.

### 3.3 Analysis of conserved motifs, domains, and gene structure in CmGLK family members

**Figure 4:**
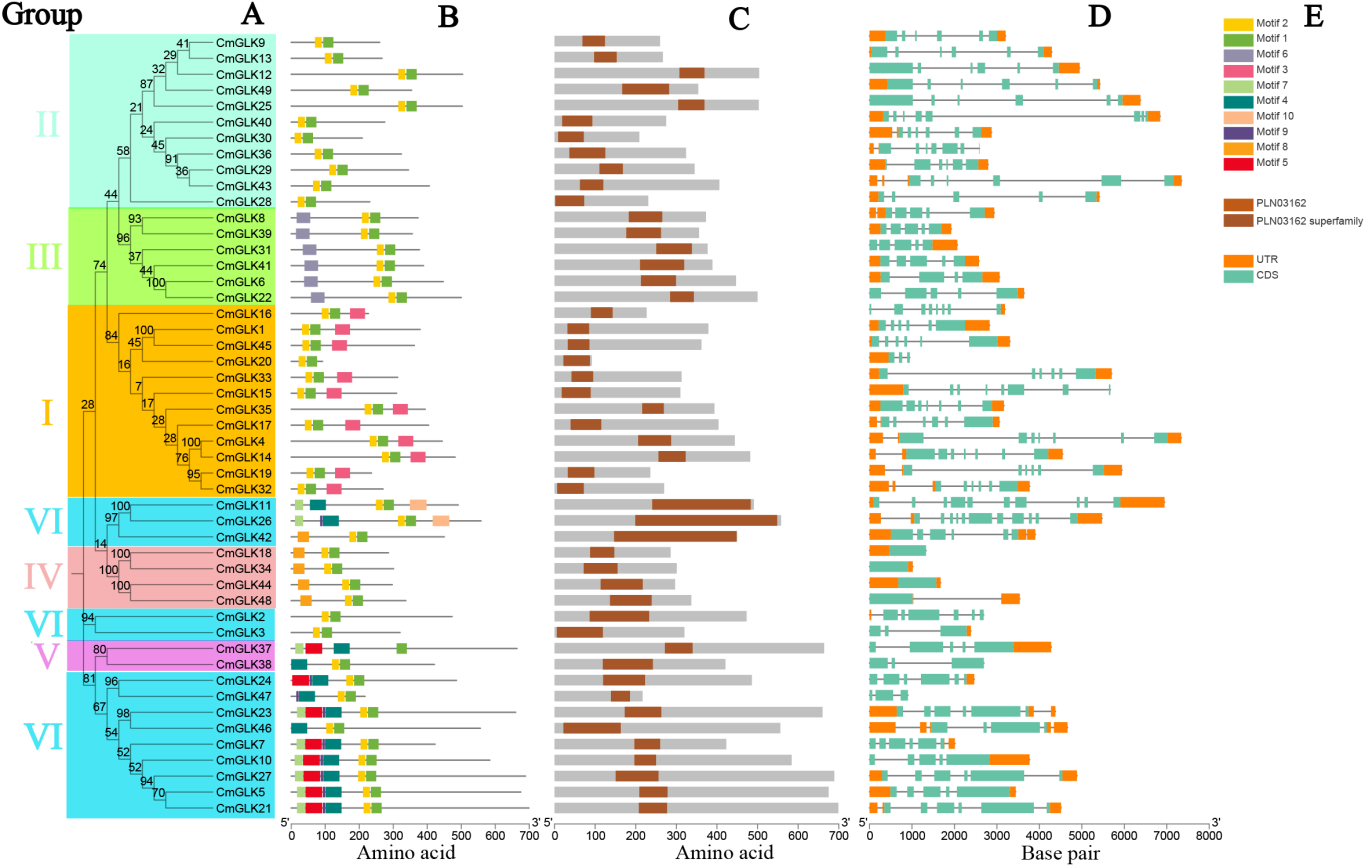
Phylogenetic tree, conserved motifs, domains, and gene structure analysis of *CmGLK* family members. **(A)** Neighbor-Joining phylogenetic tree. **(B)** Conserved protein motifs. **(C)** Distribution of *GLK* domains. **(D)** Exon and intron distribution. **(E)** Color-coded rectangles representing the types of motifs, domains, exons, and introns.

We integrated the Neighbor-Joining phylogenetic tree, conserved motifs, domains, and gene structure of *CmGLK* family members for visualization. **Figure 4A** shows the Neighbor-Joining phylogenetic tree of the *CmGLK* family members. A total of 10 conserved motifs were identified in the *CmGLK* family members. The NCBI CDD database identified Motif 1 and Motif 2 as sequences characteristic of the GOLDEN2-LIKE transcription factor. All *CmGLK* family members contained Motif 1, while the only exception was *CmGLK37*, which lacked Motif 2. The other 48 members contained Motif 2. Members within the same group exhibited highly conserved motifs in both type and number (**Figure 4B**). **Figure 4C** indicates that all *CmGLK* family members contained the PLN03162 domain (GOLDEN2-LIKE like transcription factor), confirming that these genes belong to the *GLK* type.

The gene structure of *CmGLK* family members was diverse, with variations in the number of exons and introns within the same group (**Figure 4D**). In Group I, the number of exons ranged from 3 to 8, and the number of introns ranged from 2 to 7. In Group II, exons ranged from 5 to 8, and introns ranged from 4 to 7. In Group III, the number of exons ranged from 4 to 5, and introns ranged from 3 to 4. All four *CmGLK* members in Group IV contained only 1 exon. In Group V, the number of exons ranged from 3 to 5, and introns ranged from 2 to 4. In Group VI, the number of exons ranged from 3 to 11, and introns ranged from 2 to 10. **Figure 4E** presents the color-coded rectangles corresponding to motifs, domains, and exon/intron structures. In conclusion, the diversity in protein motifs and gene structures suggests that *CmGLK* family members may have a broad range of functions in regulating the growth and development of melon.

### 3.4 Chromosomal localization, duplication, and Ka/Ks ratio of CmGLK genes

**Figure 5:**
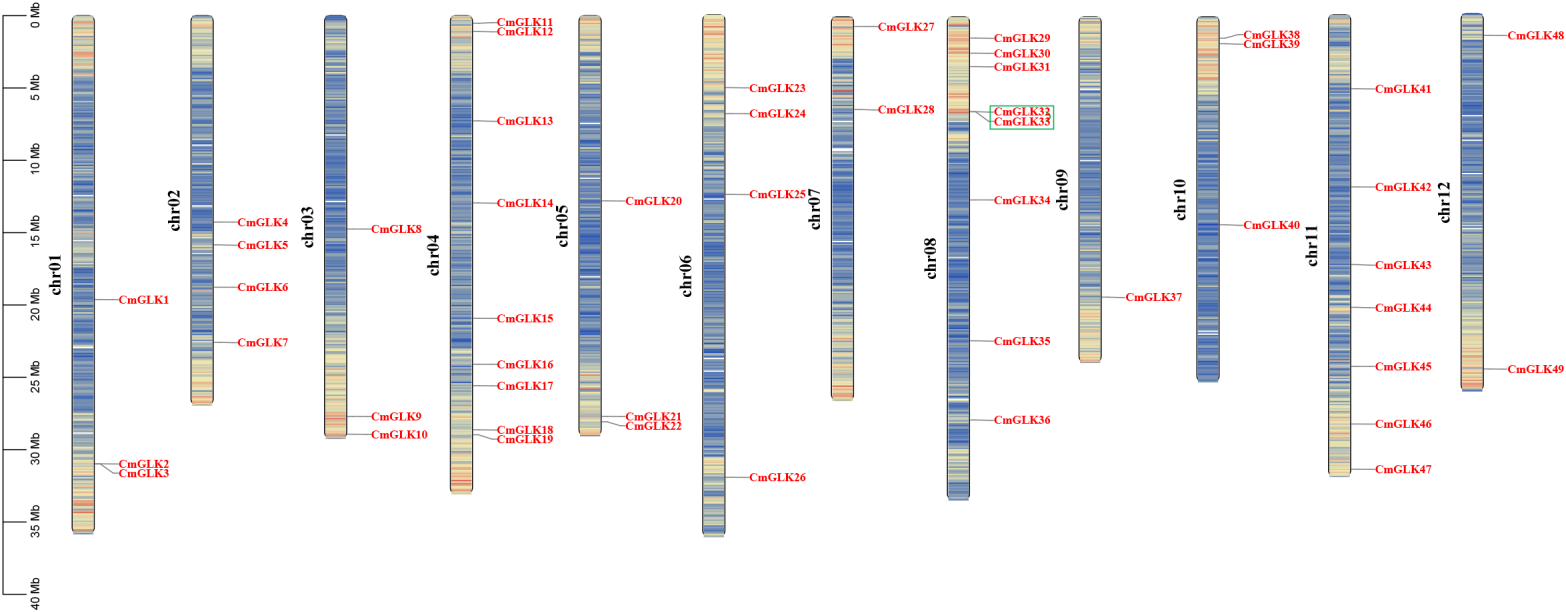
Chromosomal localization of *CmGLK* family members. The left scale represents chromosome length, with “chr” denoting chromosomes. Gene density for each chromosome was calculated at intervals of 200 kb. A color gradient from blue (low gene density) to red (high gene density) indicates gene density, while blank regions represent areas with no gene distribution information. Green boxes denote tandem duplication gene pairs.

The *CmGLK* family members were unevenly distributed across 12 chromosomes (**Figure 5**). Specifically, chromosomes chr1 to chr12 contained 3, 4, 3, 9, 3, 4, 2, 8, 1, 3, 7, and 2 *CmGLK* genes, respectively. According to the chromosomal density map, *CmGLK* family members were primarily located in regions with a high density of genes on the chromosomes.

**Figure 6:**
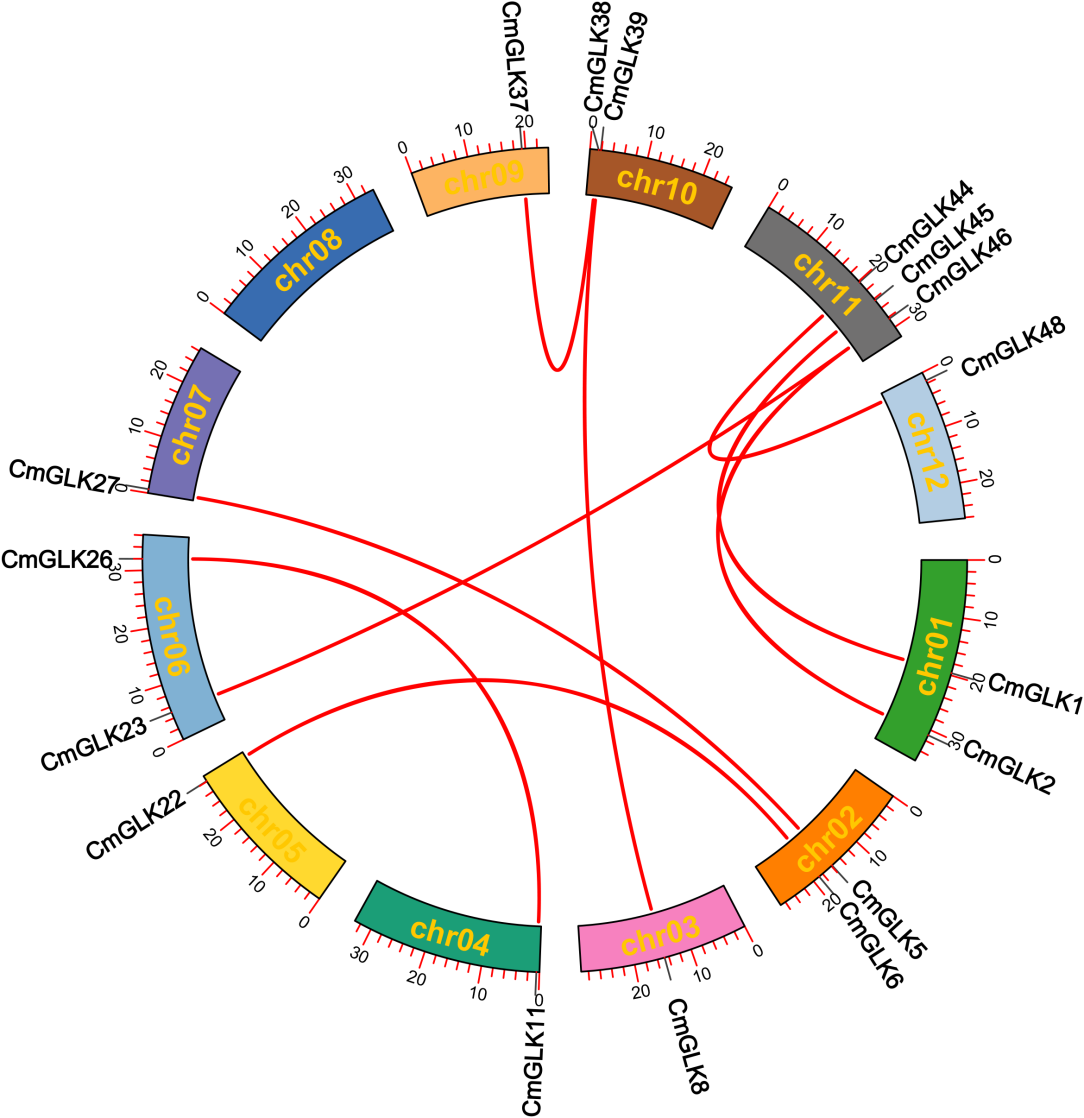
Collinearity analysis of *CmGLK* family members within chromosomes red lines represent segmental duplication gene pairs.

**Figure 7:**
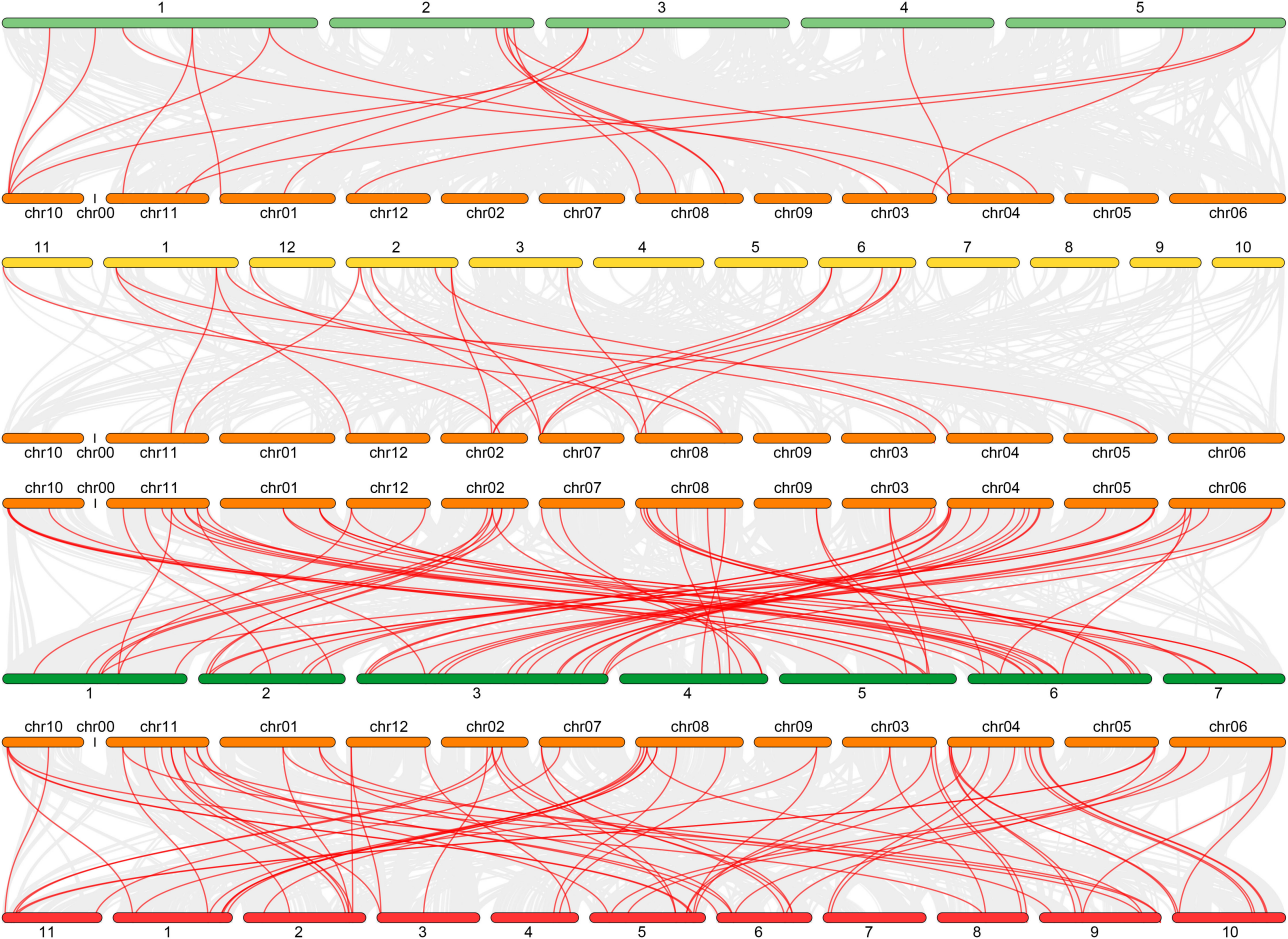
Distribution of *GLK* family homologous gene pairs. **(A)** *A. thaliana* and *C. melo*. **(B)** *O. sativa* and *C. melo*. **(C)** *C. melo* and *C. sativus*. **(D)** *C*. *melo* and *C.lanatus*. Red lines represent homologous gene pairs. Orange segments represent melon chromosomes, cyan segments represent *A*. *thaliana* chromosomes, yellow segments represent rice chromosomes, dark green segments represent cucumber chromosomes, and red segments represent watermelon chromosomes. Numbers represent the corresponding chromosome numbers.

Nine pairs of segmental duplication genes within the *CmGLK* family were identified: *CmGLK1/CmGLK45*, *CmGLK2/CmGLK46*, *CmGLK5/CmGLK27*, *CmGLK6/CmGLK22*, *CmGLK8/CmGLK39*, *CmGLK11/CmGLK26*, *CmGLK23/CmGLK46*, *CmGLK37/CmGLK38*, and *CmGLK44/CmGLK48* (**Figure 6**; **Supplementary Table S2**). Additionally, one tandem duplication gene pair, *CmGLK32/CmGLK33* (**Figure 5**), was identified. Homologous *GLK* gene pairs between melon and four other species (*A. thaliana*, rice, cucumber, and watermelon)were also identified, with 19, 19, 68, and 69 pairs of homologous genes, respectively (**Figure 7**; **Supplementary Table S2**). Notably, a high level of homology was observed between the *GLK* family members in melon, cucumber, and watermelon. Several *CmGLK* members, such as *CmGLK31* and *CmGLK39*, exhibited homology with distinct cucumber or watermelon GLK members. Finally, the majority of segmental duplication gene pairs, tandem duplication gene pairs, and homologous gene pairs between melon and cucumber, as well as melon and watermelon, exhibited Ka/Ks values of less than 1 (**Figure 7**; **Supplementary Table S2**), suggesting purifying selection. However, for most homologous gene pairs between melon and *A. thaliana*, as well as melon and rice, Ka/Ks values could not be determined.

### 3.5 CmGLK family member promoter analysis

**Figure 8:**
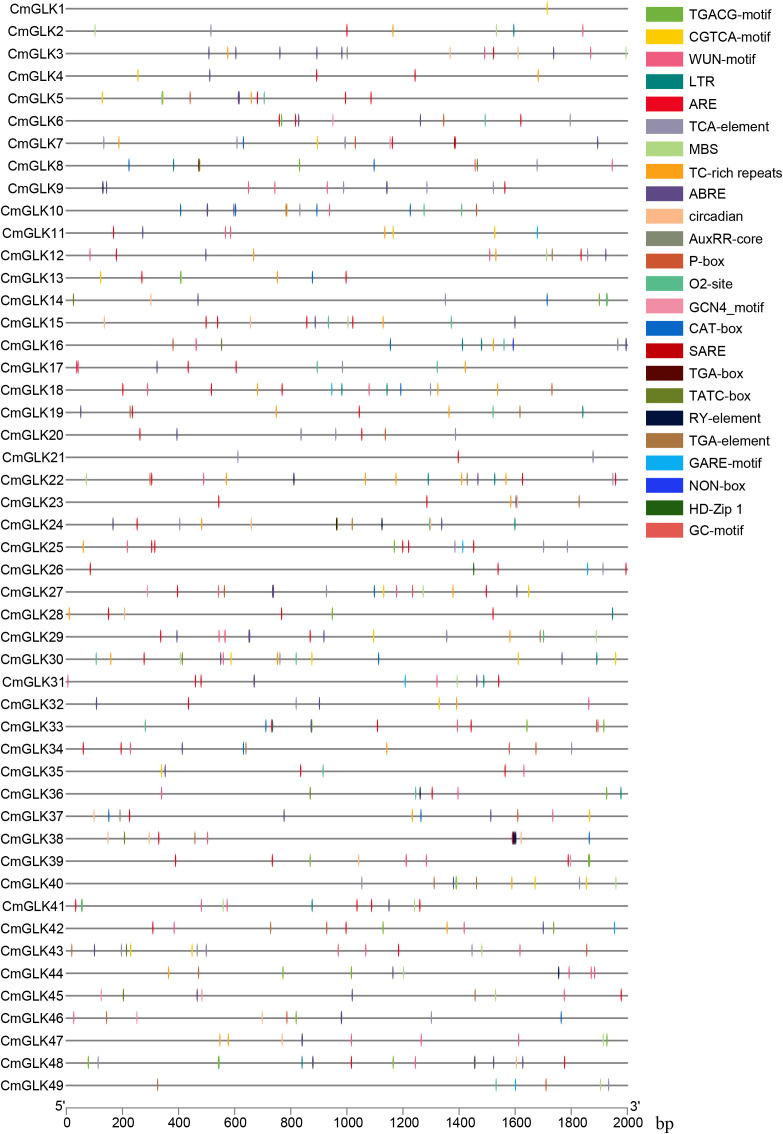
Distribution of *Cis*-regulatory elements in *CmGLK* family members.

A total of 107 types of *cis*-regulatory elements in the *CmGLK* family were identified, including numerous light-responsive elements, such as ACE, chs-CMA1a, and GT1-motif (**Supplementary Table S3**). In addition to the basic and light-related elements, these elements also include *cis*-regulatory elements associated with abiotic and biotic stresses, phytohormone responses, and plant growth and development (**Figure 8**). The abiotic and biotic stress-related *cis*-regulatory elements include six types: TC-rich repeats, LTR, ARE, GC-motif, MBS, and WUN-motif. The phytohormone response-related elements consist of eleven types: TGA-element, TATC-box, TCA-element, SARE, ABRE, AuxRR-core, TGACG-motif, CGTCA-motif, P-box, GARE-motif, and TGA-box. The plant growth and development-related elements consist of nine types:: MSA-like, circadian, RY-element, O2-site, CAT-box, NON-box, GCN4_motif, HD-Zip 1, and MBSI. In conclusion, *CmGLK* family members may stress responses.

### 3.6 Expression pattern analysis of CmGLK family members

**Figure 9:**
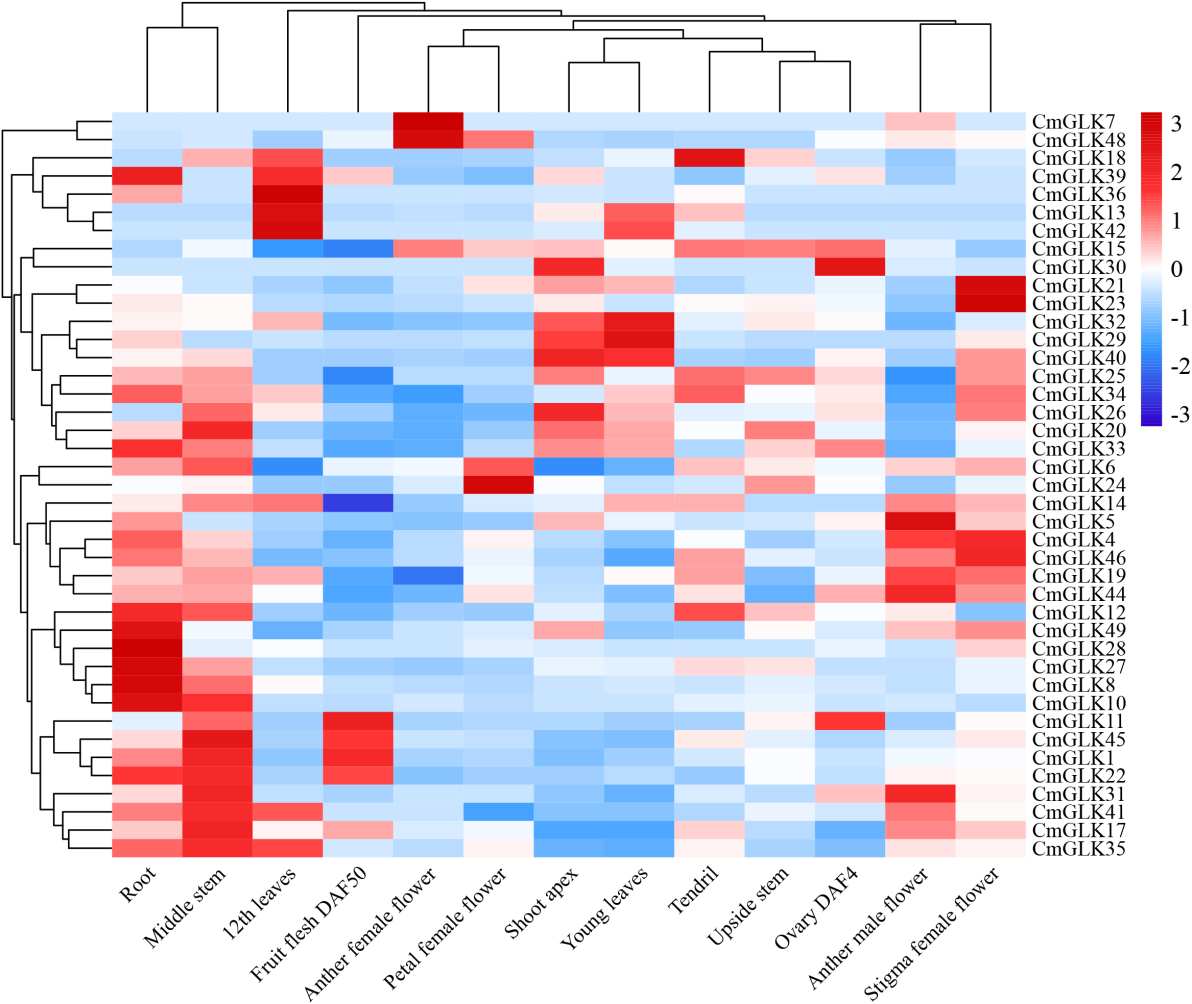
Expression heatmap of *CmGLK* family members in 13 melon tissues. The heatmap was generated using the average row normalization method. Red indicates high expression, while blue indicates low expression. Numbers on the map represent significance levels.

FPKM values for *CmGLK* family members were obtained in 13 melon tissues: root, middle stem, upside stem, shoot apex, young leaves, 12th leaves, tendril, anther male flower, another female flower, petal female flower, stigma female flower, ovary DAF4, and fruit flesh DAF50. A heatmap was generated to visualize the expression patterns (**Figure 9**; **Supplementary Table S4**). The results showed that the number of *CmGLK* genes significantly expressed was higher in root and middle stem tissues, with 15 and 16 genes, respectively. In the remaining 11 tissues, 4 to 9 *CmGLK* genes were significantly expressed. Notably, 9 genes exhibited high expression levels in the 12th leaves. Several genes exhibited tissue-specific expression patterns. For instance, *CmGLK29*, *CmGLK32*, *CmGLK36*, and *CmGLK42* were highly expressed in leaves. *CmGLK8*, *CmGLK10*, *CmGLK27*, *CmGLK28*, and *CmGLK49* were highly expressed in roots. *CmGLK8*, *CmGLK17*, *CmGLK20*, and *CmGLK26* were highly expressed in stems. *CmGLK21*, *CmGLK23*, *CmGLK24*, *CmGLK46*, and *CmGLK48* were highly expressed in flowers, most of which belong to Group VI. Among the previously identified paralogous gene pairs, five pairs (*CmGLK1/CmGLK45*, *CmGLK23/CmGLK46*, *CmGLK5/CmGLK27*, *CmGLK6/CmGLK22*, *CmGLK32/CmGLK33*) exhibited similar or identical expression patterns across tissues, suggesting that duplicated genes have retained similar evolutionary fates. These findings indicate that *CmGLK* family members exhibit significant tissue-specific expression characteristics, hormone regulation, and stress responses.

### 3.7 Quantitative fluorescence expression analysis of CmGLK family members under F. wilt stress

We used qRT-PCR to analyze the expression changes of 15 *CmGLK* genes in melon stems under *F.* wilt stress. The results (**Figure 2**; **Supplementary Table S5**) indicated that the 15 *CmGLK* genes exhibited varying degrees of expression changes at different time points after *F.* wilt inoculation. Most *CmGLK* genes exhibited a trend of increasing expression levels from 0 to 48 hours, followed by a decrease from 48h to 96h. *CmGLK1*, *CmGLK19*, *CmGLK25*, *CmGLK33*, and *CmGLK41* exhibited significantly elevated expression levels at the 24h and 48h time points, surpassing twice the expression levels of the CK group. Notably, *CmGLK33* and *CmGLK41* reached expression levels approximately 16 and 17 times higher than the CK group at 48 hours, respectively. In contrast, the remaining 10 genes exhibited relatively smaller changes in expression at the 24h, 48h, and 96h time points compared to the CK group. These results suggest that *CmGLK* family members may play regulatory roles in melon’s response to *F.* wilt stress.

### 3.8 Quantitative fluorescent expression analysis of CmGLK family members under drought stress

We used qRT-PCR to analyze the expression changes of 15 *CmGLK* genes in melon leaves under drought stress. The results (**Figure 3**; **Supplementary Table S5**) revealed varying degrees of expression changes among the 15 *CmGLK* genes at different time points after drought stress. *CmGLK25* exhibited significantly elevated expression levels at the 12h, 24h, and 48h time points, with the expression level at 24 hours reaching 21.93 times that of the CK group. *CmGLK32* exhibited expression levels twice those of the CK group at the 12h and 48h time points. The remaining 13 genes exhibited either relatively lower increases or decreases in expression levels at different time points under drought stress. These results suggest that *CmGLK* family members may play a role in enhancing the response of melon to drought stress.

## 4 Discussion

*GLK* transcription factors belong to the recently classified GARP superfamily and play crucial roles in chloroplast development, senescence, and optimizing photosynthesis under biotic and abiotic stress (Kobayashi et al., 2013; Han et al., 2016; Savitch et al., 2007; Nguyen et al., 2014). Previous studies have identified 44, 46, 66, 78, 130, 59, and 66 *GLK* genes in forsythia (Zhao Z et al., 2022), grape (Xu et al., 2022), tomato (Wang et al., 2022), moso bamboo (Wu et al., 2022), soybean (Alam et al., 2022), foxtail millet (Chen et al., 2022), and tea (Wang et al., 2024), respectively. In this study, we identified 49 *GLK* genes in the melon genome, which were classified into six groups (I–VI) based on the classification of *A. thaliana GLK* genes. Members of the same *CmGLK* subfamily exhibited highly conserved protein motifs and gene structures, confirming the reliability of the grouping. Generally, proteins within the same subgroup tend to have more similar functions. In Group II, we found three *A. thaliana KANADI* (*KAN*) genes (*AT1G32240.1*, *AT5G16560.1*, *AT5G42630.1*), which are expressed in the shoot apical meristem (SAM) and inhibit auxin biosynthesis, transport, and signaling to suppress organ morphogenesis (Ram et al., 2020). It is speculated that *CmGLK*9, *CmGLK*12, and *CmGLK*25, which belong to the same group in the phylogenetic tree, may have similar functions. Group IV included *AtLUX* (*AT3G46640.1*), *AtBOA* (*AT5G59570.1*), and *AtMYBC1* (*AT2G40970.1*), which are associated with circadian rhythm oscillations. *CmGLK18*, *CmGLK48*, *CmGLK44*, and *CmGLK34* in the same group may also perform similar functions (Zhao X et al., 2022).

Gene duplication is a crucial mechanism for gene expansion during plant genome evolution and helps plants adapt to diverse environments. Using bioinformatics techniques, we identified one tandem duplication pair and nine segmental duplication pairs in the melon genome, indicating that gene duplication significantly contributed to the expansion of the *GLK* family in melon. For example, moso bamboo has 31 segmental and 1 tandem duplication pair (Wu et al., 2022), grape has 5 segmental and 1 tandem duplication pair (Xu et al., 2022), and tea has 17 segmental and 1 tandem duplication pair (Wang et al., 2024). Segmental duplication is a primary mechanism for the expansion of the melon *GLK* family, as it has been proven to be more common than tandem duplication and plays an essential role in long-term evolution. The Ka/Ks values of segmental duplication gene pairs, tandem duplication gene pairs, and homologous gene pairs between melon and cucumber, as well as between melon and watermelon, were less than 1, suggesting strong purifying selection. These findings indicate that gene duplication and evolutionary conservation among Cucurbitaceae species contributed to the expansion of the *CmGLK* gene family.

Transcriptome data revealed FPKM values of *CmGLK* genes across 13 melon tissues. Expression profiling showed that *CmGLK* genes displayed significant and diverse expression patterns among tissues, with noticeable tissue-specific expression, which is consistent with a potential role in melon growth and development. In many plants, such as *A. thaliana*, rice, and maize, *GLK* genes are highly expressed in leaves but less so in stems, flowers (inflorescences), and roots (Fitter et al., 2010; Yeh et al., 2022), which is consistent with their central role in chloroplast development. Interestingly, our study revealed a distinct expression pattern in melon, where a considerable number of *CmGLK* genes exhibited higher expression levels in root and stem tissues. This divergence from the canonical pattern observed in model plants suggests potential neofunctionalization or subfunctionalization of the CmGLK family in melon, possibly reflecting unique physiological adaptations of this horticultural crop.”

“The biological significance of this root/stem-biased expression may be closely linked to organ-specific stress adaptation. It is established that GLK transcription factors can function beyond leaf chloroplast development. For instance, they have been documented to participate in stress response signaling pathways, sometimes independently of their photosynthetic role (Murmu et al., 2014). Moreover, the presence of functional chloroplasts in non-foliar tissues like roots, whose development can be influenced by GLK proteins, has been implicated in local energy production and stress resilience (Kobayashi et al., 2013). Therefore, we hypothesize that the high expression of specific *CmGLK* genes in melon roots could represent an adaptive strategy to enhance local defenses against soil-borne pathogens such as *F. oxysporum*, potentially by modulating redox homeostasis or energy metabolism. Similarly, their elevated expression in stems might bolster vascular function and photosynthetic capacity critical for surviving drought stress. While this compelling hypothesis requires future functional validation, it provides a rational framework for interpreting our data and underscores the functional diversity of the GLK family across plant species.”

*GLK* transcription factors have been extensively studied for their role in enhancing plant resistance to biotic stresses. Overexpression of *AtGLK1* in *A. thaliana* significantly improves resistance to *F. graminearum* (Schreiber et al., 2011) and *Botrytis cinerea* (Murmu et al., 2014). Similarly, ectopic expression of peanut *AhGLK1b* in *A. thaliana* enhances resistance to bacterial pathogen *Pst* DC3000 and *Ralstonia solanacearum* (Ali et al., 2020). The Turnip Yellow Mosaic Virus P69 interacts with *AtGLKs*, inhibiting their transcriptional activation activity, and suppressing *GLK*-targeted genes involved in light harvesting and chlorophyll biosynthesis, which affects normal plant growth (Ni et al., 2017). *GLKs* may mediate disease resistance through the SA signaling pathway. NPR1, an SA receptor, induces *SIB1* expression to initiate defense responses. *SIB1* mutants show reduced resistance to *B. cinerea*, whereas overexpression enhances resistance (Lai et al., 2011). *SIB1* interacts with *GLKs* in the nucleus to enhance transcriptional activation of *PhANGs* genes, while in chloroplasts, it interacts with SIG1 to inhibit *PhAPGs* expression, leading to the accumulation of ROS and ^1^O_2_. This accumulation activates plastid-nucleus retrograde signaling, triggering programmed cell death (PCD) and immunity (Lv et al., 2019). *LSD1* also interacts with *GLKs* to suppress their binding to target promoters, reducing transcriptional activity. Overexpression of *LSD1* suppresses *PhANGs* expression and chloroplast development, while *lsd1* mutants exhibit enhanced disease resistance (Li et al., 2021).

*F.* wilt, caused by *F. oxysporum*, is characterized by typical symptoms such as wilting and plant death. This pathogen is a soil-borne fungus that tends to occur and spread in warm, humid environments (Fravel et al., 2003).The multiple *cis*-regulatory elements in promoters play a critical role in resistance signaling pathways, and their interactions regulate the complex processes of disease resistance (Wood, 2002).The promoters of *CmGLK* genes contain various hormone response elements (**Figure 8**; **Supplementary Table S3**), including IAA (auxin) response elements (TGA-element, AuxRR-core), ABA (abscisic acid) response elements (ABRE), MeJA (methyl jasmonate) response elements (CGTCA-motif, TGACG-motif), SA (salicylic acid) response elements (TCA-element, TGA-element, SARE), and GA (gibberellin) response elements (TATC-box, GARE-motif, P-box), among others. Plant hormones not only regulate plant growth and development but also act as important signaling molecules in plant resistance responses (Bari and Jones, 2009). The interactions between these hormones can enhance the expression of resistance genes, helping plants better cope with adverse conditions. *Cis*-regulatory element analysis shows that *CmGLK1*, *CmGLK19*, *CmGLK25*, *CmGLK33*, and *CmGLK41* in the melon genome contain both methyl jasmonate and salicylic acid response elements, and their expression is significantly elevated during *F.* wilt infection. This suggests that these genes may be involved in the methyl jasmonate and salicylic acid-mediated resistance pathways, thus enhancing the plant’s resistance. Specifically, we can leverage the integrated expression data to propose more precise functional hypotheses. For instance, *CmGLK33* and *CmGLK41* not only possess these defense-related promoter elements but also exhibit particularly strong induction upon pathogen attack and are highly expressed in root tissues—the primary site of *F. oxysporum* infection. This co-localization of expression and function suggests a compelling model: we hypothesize that *CmGLK33* and *CmGLK41* act as key regulators in the root, where they are activated by JA/SA signaling pathways to coordinate downstream defense responses, potentially through modulating the expression of photosynthesis-related nuclear genes as part of a holistic defense strategy.

Additionally, the combined action of methyl jasmonate and abscisic acid can improve plant stress tolerance, as seen in RT-qPCR validation where many resistance genes in banana showed upregulated expression following ABA and MeJA treatment (Luo et al., 2023). Notably, *CmGLK*19, *CmGLK*33, and *CmGLK*41 contain both methyl jasmonate and abscisic acid response elements, indicating their potential involvement in the resistance pathways mediated by these hormones during *F.* wilt infection. Furthermore, this combined analysis approach can be extended to other stresses. The promoter of *CmGLK25*—the most responsive gene under drought stress—is enriched in ABRE elements alongside light-responsive motifs. Given its concurrent high expression in stems, we hypothesize that *CmGLK25* may function in vascular tissues to integrate ABA-dependent drought signaling with photosynthetic adjustments, thereby maintaining energy balance under water deficit. These specific, data-driven hypotheses provide clear targets for future functional validation of CmGLK genes in melon stress adaptation.

To date, extensive research has been conducted on *GLK* gene regulation in response to drought stress in various species. In maize, studies indicate that *GLK* genes may be associated with cold and drought stress responses (Liu et al., 2016). In cotton, the *GhGLK1* gene appears to play a role in drought and low-temperature stress regulation (Liu et al., 2021), with three genes showing potential negative regulation under drought stress. Significant changes have also been observed in *GhGLK46* and *GhGLK55* genes (Zhao et al., 2021). In peanuts, *AhGLK1* acts as a transcription factor that upregulates AhPORA expression during drought recovery, stimulating chlorophyll biosynthesis and photosynthesis (Liu et al., 2018). In millet, *SiGLK30*, *SiGLK38*, *SiGLK48*, and *SiGLK52* play significant roles in regulating salt, cold, and drought stress responses (Chen et al., 2022). The 15 *CmGLK* genes in melon show varied expression changes at different time points under drought stress, with *CmGLK*25 showing expression levels over 20 times higher than the CK group at 24h. Based on these findings, we hypothesize that *CmGLK* genes may contribute to the regulation of melon’s drought stress resistance.

It is also important to consider the phylogenetic framework of our study. Our classification was anchored using *A. thaliana* sequences as a reference. Given the considerable evolutionary divergence between the Brassicaceae and Cucurbitaceae families, some recently evolved, lineage-specific GLK genes in melon might not have been identified if they lack clear homologs in *A. thaliana*. Moreover, while clustering with *A. thaliana* genes provides valuable initial functional clues, the precise biological roles of the *CmGLK* genes may have diverged during evolution. Therefore, functional predictions based on these distant homologs should be interpreted with caution and require direct experimental validation in melon. Nevertheless, the strong phylogenetic conservation and synteny observed among the cucurbit species (melon, cucumber, and watermelon) confirm that our analysis provides a robust and reliable framework for studying the GLK family within the Cucurbitaceae.

In conclusion, this study provides a comprehensive genomic identification and expression atlas of the *CmGLK* gene family. It is crucial to note that the observed expression changes under stress establish a correlative, not causative, relationship. Thus, the definitive functional roles of significantly induced genes like *CmGLK25*, *CmGLK33*, and *CmGLK41* must be confirmed through future genetic experiments, such as knockout or overexpression in melon. Despite this, our work lays a solid foundation and offers a set of high-priority candidate genes. The resources and hypotheses generated here will undoubtedly guide and accelerate future functional characterization efforts aimed at enhancing stress tolerance in melon.”

## 5 Conclusion

This study identified and systematically analyzed 49 members of the *CmGLK* gene family in the melon genome (DHL92 v4.0). The analysis included their physicochemical properties, phylogenetic relationships, gene structures, protein motifs, gene duplication, and *cis*-regulatory elements in promoter regions. The *CmGLK* family was classified into six subgroups, with high conservation in gene structure and protein motifs within each subgroup. Gene duplication appears to play a critical role in the expansion of the *CmGLK* family. Expression profiling revealed tissue-specific expression patterns of *CmGLK* genes in melon and significant responses to drought and *F.* wilt stress. Collectively, our findings on tissue-specific expression and stress-induced expression changes suggest that the *CmGLK* family is likely involved in melon growth, development, and stress resistance. This study lays a foundation for future studies on the functional characterization of *CmGLK* genes and provides valuable candidates for molecular breeding efforts aimed at enhancing stress tolerance in melon.

## Data Availability

The original contributions presented in the study are included in the article/**Supplementary Material**. Further inquiries can be directed to the corresponding author.
